# Robust Multi-TE ASL-Based Blood–Brain Barrier Integrity Measurements

**DOI:** 10.3389/fnins.2021.719676

**Published:** 2021-12-03

**Authors:** Amnah Mahroo, Mareike Alicja Buck, Jörn Huber, Nora-Josefin Breutigam, Henk J. M. M. Mutsaerts, Martin Craig, Michael Chappell, Matthias Günther

**Affiliations:** ^1^MR Physics, Fraunhofer Institute for Digital Medicine MEVIS, Bremen, Germany; ^2^MR-Imaging and Spectroscopy, University of Bremen, Bremen, Germany; ^3^Department of Radiology and Nuclear Medicine, Amsterdam Neuroscience, Amsterdam University Medical Center, Amsterdam, Netherlands; ^4^Mental Health and Clinical Neurosciences, School of Medicine, University of Nottingham, Nottingham, United Kingdom; ^5^Sir Peter Mansfield Imaging Centre, School of Medicine, University of Nottingham, Nottingham, United Kingdom; ^6^Wellcome Centre for Integrative Neuroimaging, Nuffield Department of Clinical Neurosciences, University of Oxford, Oxford, United Kingdom; ^7^Nottingham Biomedical Research Centre, Queens Medical Centre, University of Nottingham, Nottingham, United Kingdom; ^8^mediri GmbH, Heidelberg, Germany

**Keywords:** blood–brain barrier (BBB), arterial spin labelling (ASL) MRI, T2 relaxation, exchange time, permeability, multi-TE ASL

## Abstract

Multiple echo-time arterial spin labelling (multi-TE ASL) offers estimation of blood–tissue exchange dynamics by probing the T2 relaxation of the labelled spins. In this study, we provide a recipe for robust assessment of exchange time (Texch) as a proxy measure of blood–brain barrier (BBB) integrity based on a test-retest analysis. This includes a novel scan protocol and an extension of the two-compartment model with an “intra-voxel transit time” (ITT) to address tissue transit effects. With the extended model, we intend to separate the underlying two distinct mechanisms of tissue transit and exchange. The performance of the extended model in comparison with the two-compartment model was evaluated in simulations. Multi-TE ASL sequence with two different bolus durations was used to acquire *in vivo* data (*n* = 10). Cerebral blood flow (CBF), arterial transit time (ATT) and Texch were fitted with the two models, and mean grey matter values were compared. Additionally, the extended model also extracted ITT parameter. The test-retest reliability of Texch was assessed for intra-session, inter-session and inter-visit pairs of measurements. Intra-class correlation coefficient (ICC) and within-subject coefficient of variance (CoV) for grey matter were computed to assess the precision of the method. Mean grey matter Texch and ITT values were found to be 227.9 ± 37.9 ms and 310.3 ± 52.9 ms, respectively. Texch estimated by the extended model was 32.6 ± 5.9% lower than the two-compartment model. A significant ICC was observed for all three measures of Texch reliability (*P* < 0.05). Texch intra-session CoV, inter-session CoV and inter-visit CoV were found to be 6.6%, 7.9%, and 8.4%, respectively. With the described improvements addressing intra-voxel transit effects, multi-TE ASL shows good reproducibility as a non-invasive measure of BBB permeability. These findings offer an encouraging step forward to apply this potential BBB permeability biomarker in clinical research.

## Introduction

Recently, the use of non-invasive MRI techniques to probe the haemodynamics across the blood–brain barrier (BBB) has gained increased interest, primarily due to the concerns arising from the contrast agent-based MRI (Barbier et al., 2002; Starr et al., 2009; Kanda et al., 2016; Olchowy et al., 2017). In comparison, water – due to its small molecule size – acts as an excellent, sensitive tracer for detecting minute and subtle damage to the BBB, which makes water-based MRI methods an ideal candidate for investigating BBB permeability (Dickie et al., 2020; Lin et al., 2021). Arterial spin labelling (ASL) sequences (Detre et al., 1992; Williams et al., 1992) have been adapted to detect the compartmentalization of the labelled water molecules, which further provide insight into the brain vascular permeability (Wang et al., 2007; Liu et al., 2011; Gregori et al., 2013; Wells et al., 2013; Lin et al., 2018; Ohene et al., 2019; Shao et al., 2020).

Most methods for perfusion quantification using ASL are based on the one-compartment Kety model (Kety, 1951), which assumes instantaneous equilibrium between the capillaries and tissue. It is recognised that this model is a simplification of the true process by which labelled blood water accumulates in the voxel (St Lawrence et al., 2000; Parkes and Tofts, 2002; Wu et al., 2010). Specifically, the model assumes instantaneous arrival of the labelled blood at the capillary exchange site in the voxel and, second, the infinite permeability of capillaries for water (Li et al., 2005). Two concepts were introduced to address the infinite exchange of water between capillaries and tissue by proposing two-compartment models and incorporating a permeability surface area product (PS) term. Zhou et al. (2001) and Parkes and Tofts (2002) assumed well-mixed concentrations of the ASL signal in the two compartments, while St Lawrence et al. (2000) proposed a distributed model that considers the change in labelled water concentration along the capillary path. However, studies (Zhou et al., 2001; Carr et al., 2007) have reported that measuring PS with conventional ASL within current hardware limitations might not be achievable and would require an increase in signal-to-noise ratio (SNR) of two orders of magnitude for ASL measurements. This problem arises because the ASL signal primarily depends on T1 relaxation, which leads to substantial decay in signal at longer post-label delay times. An alternative approach to measure the exchange dynamics has been proposed by probing the T2 magnetization of the blood and tissue (Li et al., 2005; Wells et al., 2009; Liu et al., 2011; Gregori et al., 2013; Ohene et al., 2021). Gregori et al. (2013) developed a T2-based two-compartment model and incorporated an exchange time term reflecting the rate at which the labelled water moves from the blood into the tissue. Such a parameter could be used to detect the slow and subtle changes in the BBB, which occur at an early stage of neurovascular diseases (van de Haar et al., 2016; Sweeney et al., 2018) and could potentially be developed into a non-invasive BBB imaging biomarker.

In this work, we have extended the two-compartment model (Gregori et al., 2013) by incorporating an additional delay time to account for transit within the voxel, beyond the usually defined arterial transit time (ATT) for the label to reach the voxel (Alsop and Detre, 1996; Barbier et al., 2001; Wang et al., 2002). This delay is called “intra-voxel transit time” (ITT) and represents the time it takes for the labelled water to travel through the arteries and arterioles before it reaches the capillary bed for exchange (Li et al., 2005; Liu et al., 2011; Wells et al., 2013). ASL-subtracted data may be regarded as a signal originating from the perfused tissue, but in reality, the imaging voxel also contains contributions of intravascular regions corresponding to smaller arteries and arterioles. These vessels further branch out to become a capillary network where exchange takes place. The main goal of introducing an additional delay time was to separate the two distinct mechanisms of tissue transit and exchange. These two phenomena have their distinct properties and are expected to fluctuate distinctively. The tissue transit time is a physiological parameter and is expected to be influenced by factors like changes in perfusion, blood flow velocities and the diameter of the vessels. We assume that this parameter has a capacity to fluctuate more strongly as compared to the exchange time, which characteristically is a structural parameter determined by the integrity and permeability of the vessels. We assume this parameter not to vary largely in healthy humans and to only change due to pathology or known aging factors. When ITT is not accounted for, we might measure an apparent exchange time that could be a combination of these two mechanisms and could suppress the actual changes in the BBB, leading to low sensitivity in detecting pathologies.

Here, we present a multi-TE ASL-based technique for the evaluation of the BBB integrity with an extended model accounting for within-voxel transit time. We compare the extended model with the two-compartment model (Gregori et al., 2013), using simulations and *in vivo* data. Additionally, we evaluate the test-retest reproducibility of the method, yielding whole-brain BBB integrity maps in healthy humans, which to our knowledge is the first multi-TE ASL study.

## Extended Model Theory

The labelled water is delivered to the voxel *via* the arteries. The existing model presented by Gregori et al. (2013) assumes that it immediately arrives in the capillary bed, and exchange of labelled water into the extravascular space takes place. However, the labelled water may take some time to traverse the arterioles, with negligible exchange (Alsop and Detre, 1996; Barbier et al., 2001; Wang et al., 2002).

In the extended model, this time is accounted for by incorporating the ITT delay. This modification addresses the limitation of the instantaneous arrival of the labelled water in the capillary bed as soon as it reaches the imaging slice, which may not be true (Wong et al., 1997; Barbier et al., 2001; Wang et al., 2002; He et al., 2012; Wells et al., 2013; Buck et al., 2021). With the ITT addition, we assume that the transit within the voxel and the exchange time are governed by two different underlying mechanisms where the former is a physiological parameter while the latter is a structural one.

In the extended model, the total measured signal is separated into three components. The first component (*S*_bl1_) defines the signal coming from the arteries in the imaging voxel. These arteries further branch into arterioles and capillaries. The labelled blood travels through these smaller vessels and ultimately reaches the capillary exchange site. The signal coming from the labelled blood within the capillary (intravascular space) forms the second component (*S*_bl2_), and the labelled blood that exchanges into the tissue (extravascular space) forms the third component (*S*_ex_). The total signal measured is the sum of all three components.

The main modification is the addition of the first component where the labelled blood is assumed to decay with T1 relaxation of the blood for the duration of ITT. The signal of the first component (*S*_bl1_) is calculated by the following:

(1) $$S_{\mathrm{bl1}}\,\left(\text{TI}\right)=2\,M_{0}\,f\,\int_{0}^{T\,I}c\,\left(t\right)\cdot m_{\text{bl}}\,\left(\text{TI}-t\right)\,dt-2\,M_{0}\,f\,\int_{0}^{T\,I}c\,\left(t-\mathrm{ITT}\right)\cdot m_{\text{bl}}\,\left(\text{TI}-t+\text{ITT}\right)\,d\,t$$

where *f* is perfusion, *M_0_* is the arterial longitudinal equilibrium magnetization, *c*(*t*) is the arterial input function and *m*_*bl*_(*t*−*t*′) is the magnetization relaxation function of blood. We have used the term inflow time (TI) as a convention to refer to the time points for the ease of solving equations, where TI is defined as the time from the start of the labelling to the readout. In relation to post-labelling delay (PLD), TI here is calculated as a sum of PLD and sub-bolus duration (SBD). In *S*_*bl1*_, subtraction is used to ensure that all blood flows through the first component into the intravascular and extravascular space.

The second (*S*_*bl2*_) and third (*S*_ex_) components are similar to the equations in the two-compartment model (Gregori et al., 2013):

(2) $$S_{\mathrm{bl},2}\left(\text{TI}\right)=2M_{0}f\int_{0}^{T\,I}c\left(t-\text{ITT}\right)\cdot r_{\text{blex}}\left(\text{TI}-t+\text{ITT}\right)\cdot m_{\text{bl}}\,\left(\text{TI}-t+\text{ITT}\right)\,d\,t$$

(3) $$S_{\text{ex}}\left(\text{TI}\right)=2M_{0}f\int_{0}^{T\,I}c\left(t-\text{ITT}\right)\cdot \left[1-r_{\text{blex}}\left(\text{TI}-t+\text{ITT}\right)\right]\cdot m_{\text{ex}}\,\left(\text{TI}-t+\text{ITT}\right)\,d\,t$$

Here, *m*_*bl*_*(t)* is the magnetization relaxation function for blood, *m*_*ex*_(*t*) is the magnetization relaxation function for tissue and additionally a blood–tissue exchange function *r*_blex_(*t*) is added. It describes the exponential blood water dynamics between the intravascular and extravascular space. It is defined as $r_{\text{blex}}=\text{exp}\,\left(-\frac{t}{T_{\text{exch}}}\right),$ where *T*_*exch*_ describes the time taken by labelled spins to exchange *via* the BBB.

For the determination of *T*_*exch*_, a multi-TE ASL sequence is used. When the magnetization of the labelled blood is flipped into the transverse plane, it starts to decay with the T2-relaxation. It is important to consider that the flipping pulse is locally played out in the imaging voxel, which means that the signal only in the voxel “sees” the flip and only this signal would be measured in the multi-TE measurements. With this condition, several cases were considered depending on the ATT, which includes the information about the amount of labelled blood that has entered the imaging voxel. These different time cases are determined based on the temporal duration of the labelled bolus.

**FIGURE 1:**
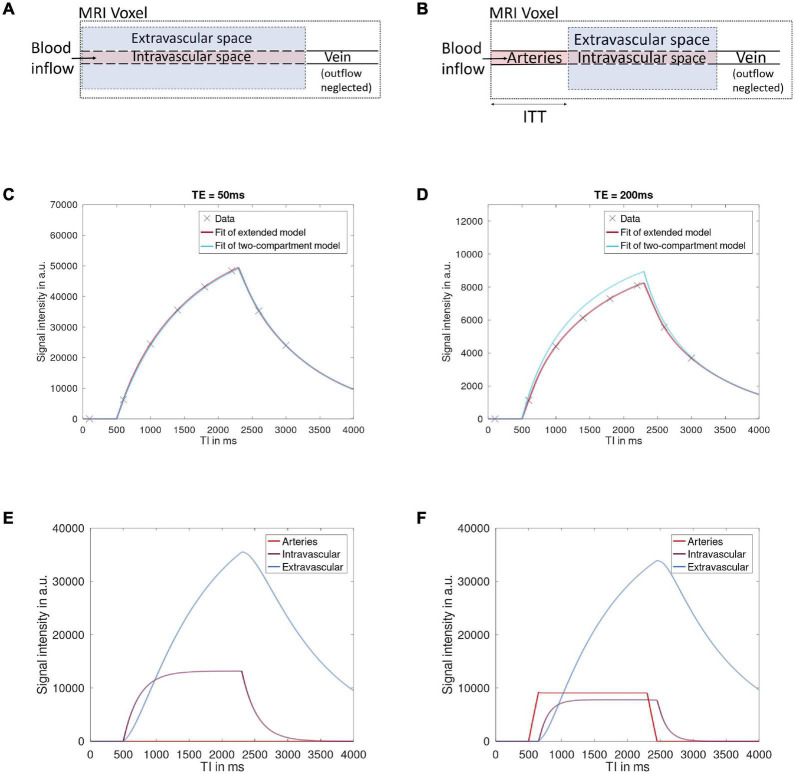
The schematic structure of the MRI voxel is shown as assumed by the two-compartment model **(A)** and the extended model **(B)**. In the extended model, it can be seen that the signal from the arteries is also included as an additional component. After arrival in the voxel, the labelled blood flows through the arteries during the ITT to the capillary exchange site where exchange of the labelled blood between the vessel (intravascular space) and tissue (extravascular space) takes place. Signal curves for data simulated at TE = 50 ms **(C)** and TE = 200 ms **(D)** are shown. The two models generate nearly identical signal curves at a lower TE, but at a higher TE value, the two-compartment model could not properly reproduce the signal. The composition of the total signal from the individual components resulting from the two-compartment model **(E)** and the extended model **(F)** is shown.

The inflow curves resulting from the two models and their respective components are shown in Figure 1. Data was simulated with ITT = 200 ms, Texch = 150 ms, ATT = 500 ms, bolus duration = 1,800 ms and two different values of TEs, 50 and 200 ms. Figure 1C shows fitted results for TE = 50 ms resulting from the two models. The resulting signal curves are nearly identical, and the Texch values estimated by the two-compartment model and the extended model were 273 and 150 ms, respectively. The extended model efficiently recovers the simulated Texch value, but the two-compartment model overestimates Texch by 82% relative to the simulated value. In Figure 1D, the signal curves resulting from the two models at TE = 200 ms are shown. It was observed that for higher TE values, the two-compartment model could not reproduce the signal curve properly. Figures 1E,F show the comparison of the individual components of the total signal (shown in Figure 1C) resulting from the two-compartment model and the extended model, respectively. For the two-compartment model (Figure 1E), it can be clearly seen that the signal in the arteries is zero, as this component is not included in the model. At ATT = 500 ms, the signal is already in the intravascular and extravascular space as exchange is assumed to take place as soon as the labelled blood arrives in the voxel. On the contrary, the signal in the extended model at ATT = 500 ms (Figure 1F) is initially only in the arteries, and only after the additional ITT the signal appears in the intravascular and extravascular space.

## Materials and Methods

### Simulated Experiments

To assess the performance of the extended model, data with a matrix size of 100 × 100 × 3 was simulated for two multi-TE pCASL protocols using MATLAB. Supplementary Table 1 shows the parameters used to simulate the data. The first dataset was generated with a short bolus duration of 450 ms yielding seven TIs (ms): 600, 1,000, 1,400, 1,800, 2,200, 2,600, and 3,000. The second protocol was generated with a longer bolus of 1,050 ms with three TIs (ms): 1,600, 2,600 and 3,600. Each dataset was generated with eight TE values (ms): 13.84, 41.52, 69.2, 96.88, 124.56, 152.24, 179.92, and 207.6. The total vascular water transit time is reported to be around 3–4 s (Ibaraki et al., 2007; He et al., 2012). The range of TIs was selected to cover this duration effectively with a combination of PLD and labelling duration to get an optimal signal. Both datasets were generated with ATT in the range of 500 ≤ ATT ≤ 2,500 ms; Texch in the range of 10 ≤ Texch ≤ 1,000 ms and a fixed ITT of 200 ms. Perfusion was fixed at 60 ml/100 g/min. To evaluate the influence of ITT, an additional dataset in the range of 100 ≤ ITT ≤ 500 ms was generated. The range of ATT was selected to cover the reported values in healthy grey matter and cerebrovascular diseases (Alsop et al., 2015). The exchange time values reported in the literature varies. PET data (Raichle et al., 1983) was taken as a reference, and a range was selected to cover the reported values in the human brain (Alsop and Detre, 1996; Wang et al., 2002; Li et al., 2005; Liu et al., 2011).

The two-compartment model and the extended model were incorporated into the FSL FABBER non-linear fitting framework based on Bayesian inference (Chappell et al., 2008). Two estimation routines were carried out for each dataset to compare the two-compartment model (Gregori et al., 2013) with the extended model. The first routine was done with a fixed ITT = 0, which ignores the tissue transit time as assumed in the original two-compartment model. In the second routine, ITT was also inferred from the data along with the other parameters [cerebral blood flow (CBF), ATT and Texch]. Percentage deviation error of the parameter estimates was calculated relative to the true value for comparison. Identity line plots were generated to visually inspect the difference between the true and estimated values.

### *In vivo* Experiments

All experiments in this study have been approved by the Ethical Committee of the University of Bremen, Bremen, Germany. Written informed consent was obtained before the participants were enrolled in the study.

### MR Imaging

**FIGURE 2:**
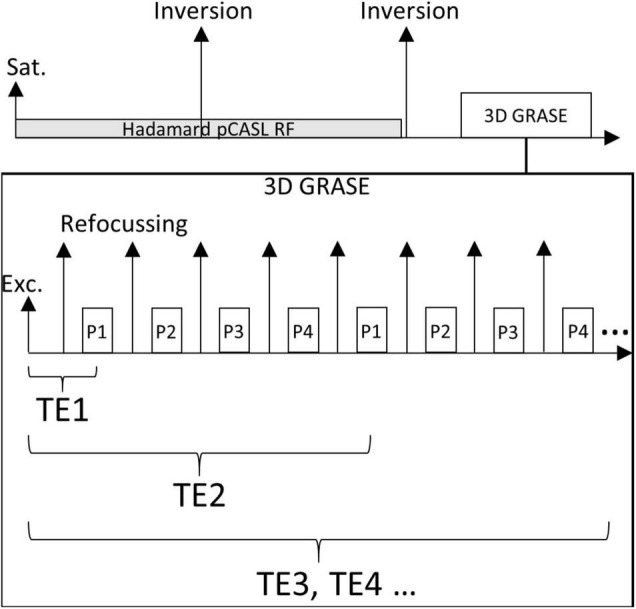
A schematic diagram of the Hadamard multi-TE ASL sequence. After presaturation, two inversion pulses are played out during the Hadamard labelling train and the post-labelling delay for background suppression. Using 3D GRASE, multiple k-space partitions as well as echo times are acquired after a single excitation. Here, an example using four partitions (P1–P4) is shown.

Ten healthy volunteers (four females, aged 28–40 years) were examined at 3T (MAGNETOM Skyra, Siemens Healthineers AG) using a 20-channel head coil. A multi-TE Hadamard pseudo-continuous (pCASL) sequence (Günther, 2007; Schmid et al., 2015) with 3D GRASE readout (Günther et al., 2005) was used. The sequence diagram is shown in Figure 2.

Two protocols of multi-TE pCASL with different bolus durations were used to acquire data. In a previous abstract (Mahroo et al., 2021), we presented that a combination of two different bolus lengths improves the sampling times resulting in a better estimation of Texch parameter. The first protocol was acquired using a Hadamard-8 matrix with a SBD of 400 ms and a PLD of 200 ms. The resulting seven TIs (ms) were 600, 1,000, 1,400, 1,800, 2,200, 2,400, and 3,000. The second protocol was acquired using a Hadamard-4 matrix with a sub-bolus duration of 1,000 ms and a PLD of 600 ms. The resulting three TIs (ms) were 1,600, 2,600 and 3,600. Both protocols were acquired at eight echo times [TE (ms)]: 13.84, 41.52, 69.2, 96.88, 124.56, 152.24, 179.92, and 207.6. Background suppression was achieved by two FOCI inversion pulses, timed to suppress T1 values of 700 and 1,400 ms, 100 ms before the imaging readout. All pCASL measurements were acquired with TR = 5,000 ms, matrix size = 64 × 128 × 24, voxel size = 2.5 × 2.5 × 5 mm^3^, FOV = 320 mm, FOV phase = 50%, resolution = 2.5 × 2.5 × 5 mm^3^, turbo factor = 2, EPI factor = 16, bandwidth = 2,298 Hz/Px and GRAPPA 2 × acceleration. M0 images were acquired to quantify the perfusion values using long TR approach (TE: 11.6 ms; TR: 5,000 s). T1 MPRAGE images were acquired for registration to the structural data.

To evaluate the reliability of the multi-TE pCASL technique, we scanned each participant on two different days, with an inter-scan interval of at least 1 week, and assessed the intra-session and inter-session test-retest reproducibility. On each visit, the participant went through two identical sessions with a short break (10 min) in between and were repositioned between the sessions. Within each session, both multi-TE pCASL protocols were acquired twice, back-to-back, which gave an evaluation of the intra-session reproducibility. The total scan duration per day was 45 min.

### Data Analysis

#### Study 1: Comparison of Extended Model

Data was analysed using the Oxford Centre for Functional MRI of the Brain (FMRIB)’s software library (FSL). The structural data was processed using the fsl_anat pipeline. Whole-brain masks and grey and white matter probability maps were used to mask the voxels of interest. The encoded ASL time series were motion corrected with the FSL MCFLIRT tool with a six-parameter rigid transformation. The ASL signal at each TE and TI was decoded by applying the respective Hadamard scheme. The resulting decoded images from the two protocols were concatenated. The final dataset was then fitted voxel-wise within the whole brain mask using the two-compartment model and the extended model for comparison. The two-compartment model fitted three parameters: ATT, perfusion and exchange time. Perfusion images from both models were quantified using the voxel-wise method (Pinto et al., 2020). The extended model additionally fitted the ITT parameter. *Post hoc* smoothing with a × 2 voxel size kernel (FWHM) was applied. All the resulting parameters were registered to structural image, and grey and white matter masks were applied to estimate the subject-specific mean parameter values. To visually compare results across the subjects, the parameter maps were transformed to standard MNI space (2 mm brain). The percentage differences of the mean grey matter values of the estimated parameters, between the two models were compared relative to the two-compartment model. A two-tailed independent *t*-test was used to evaluate the significance (*P* < 0.05) of differences across subjects. The two models were compared using the negative free energy of the model fit. The free energy provides the Bayesian evidence for a model and accounts for both goodness of fit and penalty for the number of free parameters in the model. The smaller the value of free energy, the better the model is at explaining the data (Chappell et al., 2008).

#### Study 2: Test-Retest Reproducibility

The reproducibility design resulted in eight sets of estimated parameters (CBF, ATT, ITT, and Texch). The statistical analysis for all the parameters was performed to assess intra-session, inter-session and inter-visit reproducibility using three statistical metrics. The test-retest reproducibility of all estimated parameters was assessed by intra-class correlation coefficient (ICC). Bland–Altman plots were generated to visualise the reliability and spread of the fitted parameters. The coefficient of variance (CoV) was computed to assess the precision of the method.

## Results

### Simulated Experiments

**FIGURE 3:**
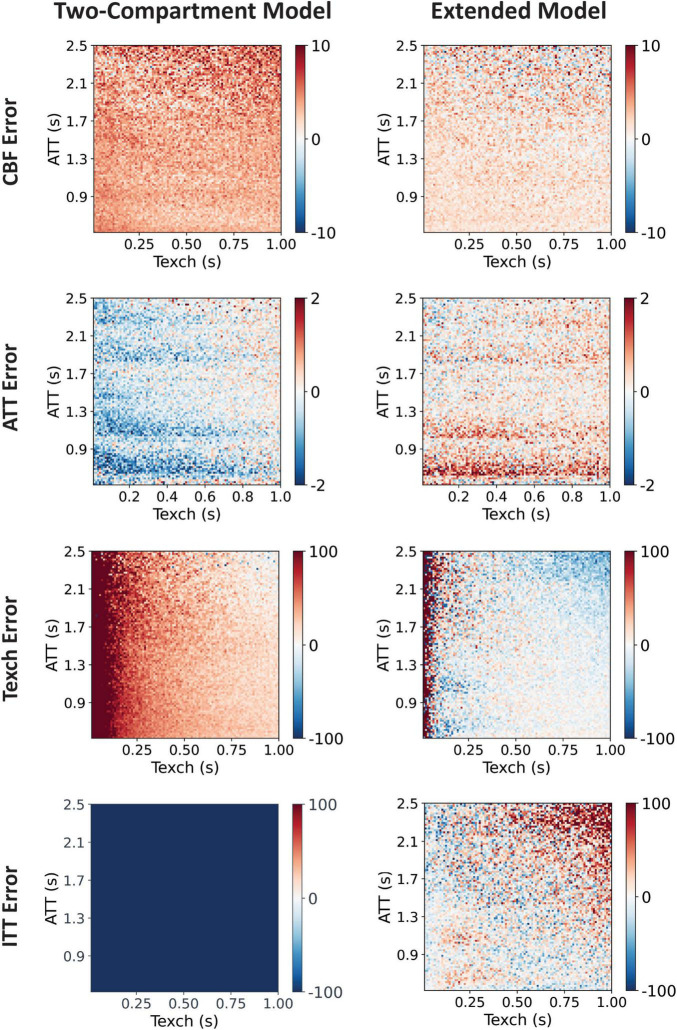
Comparison of the two-compartment model and the extended model. The data was simulated with ITT = 200 ms and was fitted with both models, and error in terms of percentage deviations of the estimated parameters was calculated. Here, errors from all four parameters resulting from the two models are displayed. It can be observed that the two-compartment model assuming no intra-voxel transit yields more error in the estimate of all parameters. Moreover, Texch parameter is overestimated for the whole range of real Texch and even more for the lower values (<300 ms). In ATT error, streaks of error appear at the TI values used because there was no partial bolus delivery between the two consecutive TIs.

**FIGURE 4:**
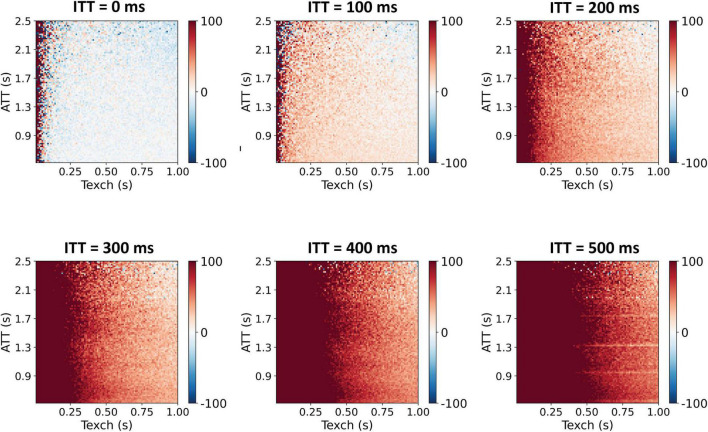
Texch percentage deviation error resulting from the two-compartment model. Test cases were generated with variable ITT values. As can be seen, even a small ITT value of 100 ms results in an overestimation of Texch, especially for the lower Texch values. The estimated apparent Texch values may correspond to a sum of the underlying true Texch and true ITT and thus result in an overestimation of the Texch parameter.

**FIGURE 5:**
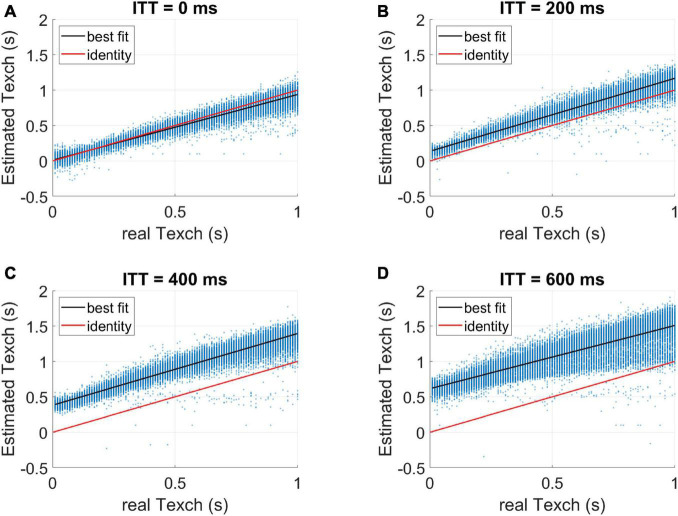
Identity line plots for Texch parameter estimated with the two-compartment model. Test cases are generated with different values of ITT. **(A)** The two-compartment model estimates Texch well when no ITT is assumed (ITT = 0 ms). All other test cases, **(B–D)**, show over-estimation of Texch with an offset approximately equal to ITT values.

The comparison of parameter errors in terms of percentage deviations relative to the true values, from the two models, are shown in Figure 3. For ITT = 200 ms, the two-compartment model resulted in higher errors for all three parameters. For ITT = 0, the comparison showed that the two-compartment model estimates Texch well when no intra-voxel transit is considered in the simulated data. The extended model, on the other hand, showed slight underestimation of lower Texch values (Supplementary Figure 6). In Figure 4, the Texch error estimates resulting from the two-compartment model, for the test cases generated with a range of ITT (ms) = 0, 100, 200, 300, 400, and 500, are shown. As mentioned earlier, the two-compartment model efficiently estimates Texch when no ITT is assumed (ITT = 0). Conversely, the data generated with as low as 100 ms of ITT leads to a small error, resulting in the overestimation of Texch. Moreover, the lower Texch values (<300 ms) show more error than the higher Texch values. Figure 5 shows the identity line plot for the Texch parameter for test cases generated with ITT (ms) = 0, 200, 400, and 600, fitted with the two-compartment model. The results of Texch error estimates and identity line plots from the extended model can be found in Supplementary Figures 3, 4, respectively.

### *In vivo* Experiments

#### Study 1: Comparison of Extended Model

**FIGURE 6:**
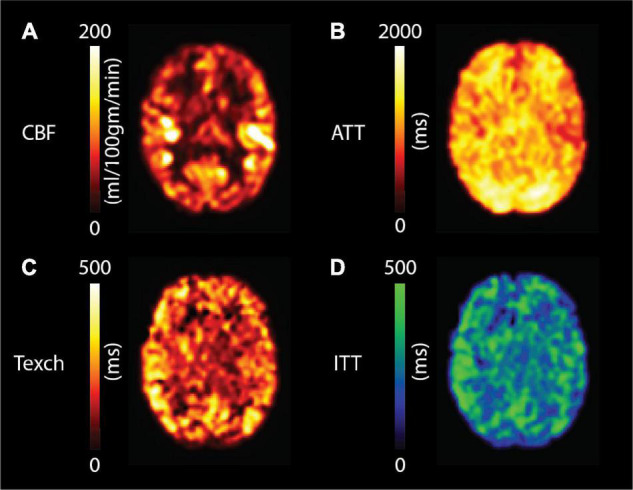
Estimated **(A)** CBF, **(B)** ATT, **(C)** Texch and **(D)** ITT maps from a representative subject in standard MNI space (middle slice).

**FIGURE 7:**
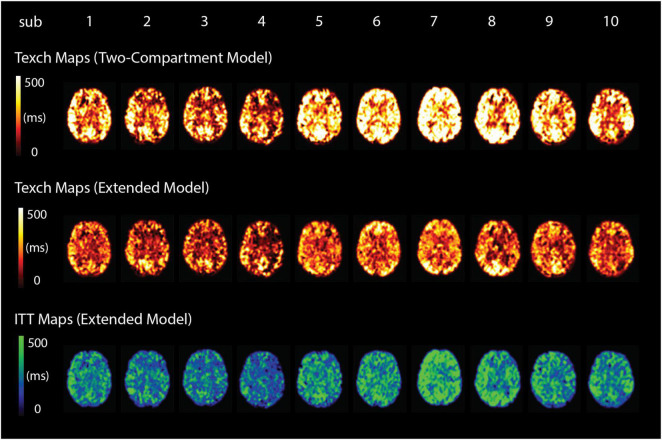
Texch and ITT whole-brain parameter maps from all 10 subjects in standard MNI space (middle slice). It can be seen that the two-compartment model estimates comparatively higher values of Texch.

Figure 6 shows the resulting estimated parameter maps of CBF, ATT, Texch, and ITT from a representative participant. Figure 7 shows the fitted Texch maps from all 10 participants estimated using the two-compartment model and the extended model. The estimated ITT maps are also shown in Figure 7. It can be visually inspected that the two-compartment model estimated higher Texch values as compared to the extended model.

**FIGURE 8:**
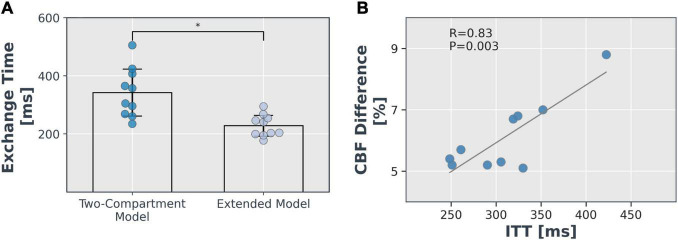
Comparison of models. **(A)** Mean grey matter exchange time with associated error (standard deviation) estimated with the two models (*P < 0.05). **(B)** The correlation of perfusion difference estimated with the two models relative to two-compartment model and ITT parameter.

The mean grey matter Texch was estimated to be 32.6% lower by the extended model (227.9 ± 37.9 ms) relative to the two-compartment model (342.0 ± 85.3 ms, *P* = 0.002, Figure 8A). This implies that the Texch measured with the two-compartment model could be a combination of real Texch and transit effects and may produce overestimated values. The difference of the estimated parameters between the models, relative to the two-compartment model, is summarised in Table 1. The mean difference between CBF and ATT was 6.1 ± 1.2%, *P* = 0.435 and 0.8 ± 0.4%, *P* = 0.908, respectively, and was not significant. The extended model additionally estimated the ITT parameter, and the mean grey matter ITT was 310.43 ± 59.2 ms, averaged across the subjects.

**TABLE 1:** Relative difference of estimated CBF, ATT, and Texch between the models, relative to the two-compartment model.

Sub	CBF (%)	ATT (%)	Texch (%)
1	5.2	−1.0	32.6
2	5.2	−0.3	24.5
3	5.7	−0.3	25.1
4	5.4	−0.1	24.4
5	5.3	−1.2	32.6
6	5.1	−1.2	36.9
7	8.8	−1.1	41.7
8	7.0	−0.9	37.5
9	6.8	−0.9	32.7
10	6.7	−0.7	33.2
Mean	6.1	−0.8	32.1
*P*-value	0.435	0.908	0.002

The results of mean grey matter negative free energy are shown in Supplementary Table 5. The extended model did not appear to offer an improvement over the two-compartment model, but on average offered a similar performance.

Moreover, it was observed that the perfusion difference between the two models significantly correlated with the ITT value (*r* = 0.83, *P* = 0.003) estimated with the extended model (Figure 8B). This implies that the brain regions with a higher ITT showed more difference in the CBF estimated with the two models.

#### Study 2: Test-Retest Reproducibility

Table 2 shows the summary of ICC and CoV results of all the estimated parameters. The intra-session CoV of Texch, CBF, ATT and ITT were 6.6%, 3.4%, 1.7%, and 2.1%, respectively, reflecting good reliability of all the estimated parameters. For further analysis of reproducibility, the encoded ASL data from the intra-session measurements (back-to-back) were averaged to improve the SNR. The resulting averaged data was used to compute inter-session and inter-visit reliability. It was observed that the CoV of Texch for inter-session and inter-visit was 7.9 and 8.4%, respectively, suggesting that Texch estimation based on the proposed measurement is reliable, and relevant sources of fluctuations have been addressed.

**TABLE 2:** Reproducibility results of intra-session, inter-session and inter-visit ICC and CoV of the mean grey matter estimates for all parameters estimated with the extended model.

Parameter	Session	ICC	*P*-value	% CoV
Texch	Intra-session	0.781	0.001	6.6
	Inter-session	0.719	0.008	7.9
	Inter-visit	0.566	0.032	8.4
Perfusion	Intra-session	0.954	<0.001	3.4
	Inter-session	−0.043	0.546	11.0
	Inter-visit	−0.692	0.972	14.2
ATT	Intra-session	0.973	<0.001	1.7
	Inter-session	0.952	<0.001	2.4
	Inter-visit	0.915	<0.001	3.2
ITT	Intra-session	0.979	<0.001	2.1
	Inter-session	0.891	<0.001	4.6
	Inter-visit	0.806	<0.001	6.0

**FIGURE 9:**
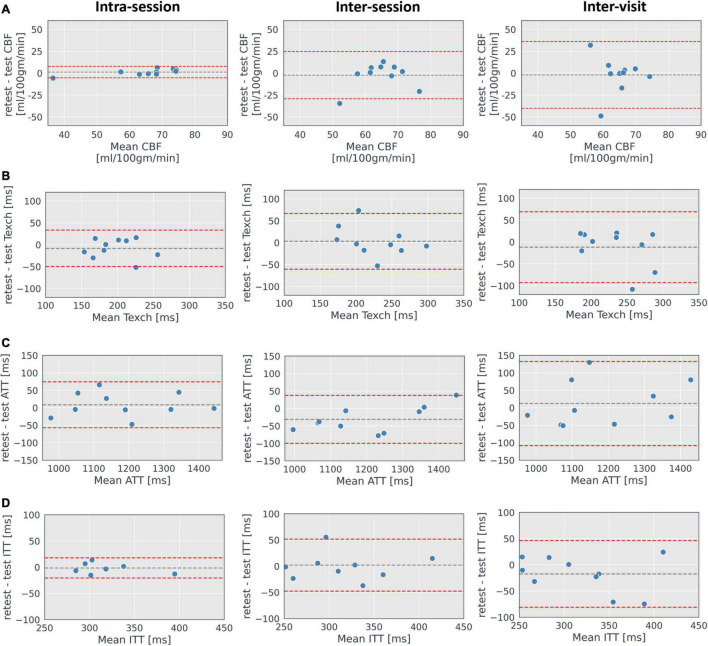
Bland–Altman plots. Plots of mean grey matter CBF **(A)**, Texch **(B)**, ATT **(C)**, and ITT **(D)** showing the spread of the data for intra-session, inter-session and inter-visit estimated with the extended model. Grey dashed line shows the mean difference, and red dashed lines represent 1.96 × standard deviations of the mean, displaying the corresponding limits of agreement.

**FIGURE 10:**
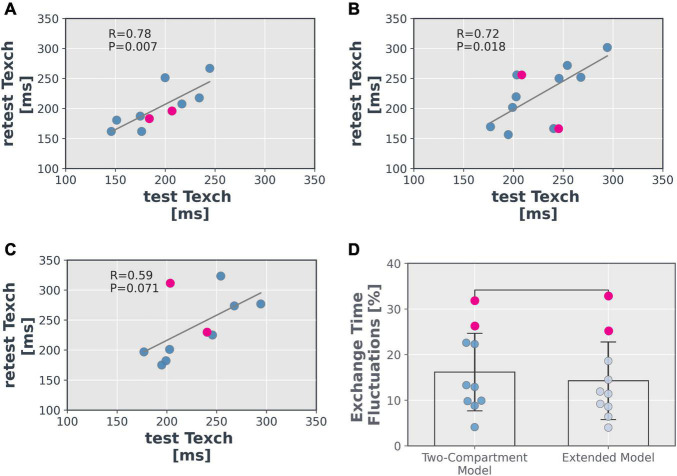
Scatter plots of mean grey matter Texch from **(A)** intra-session, **(B)** inter-session and **(C)** inter-visit using the extended model. **(D)** Fluctuations measured within the Texch values estimated by the models in terms of the normalised difference between the two visits (*P* = 0.321). Inter-session data from two subjects showed a notably low ASL signal (difference of a factor of 2) in the decoded images possibly due to improper shimming or inefficient labelling and is shown in pink data points.

The ICC results for all the parameters were significant except for inter-session CBF and inter-visit CBF (ICC = −0.04, *P* = 0.55 and ICC = −0.69, *P* = 0.97, respectively). Figure 9 shows the Bland–Altman plots of CBF, Texch, ATT, and ITT for the intra-session, inter-session and inter-visit repeatability. The mean intra-session, inter-session and inter-visit values of Texch along with other estimated parameters are shown in Table 3. Scatter plots of Texch for inter-session, intra-session and inter-visit are shown in Figure 10. Scatter plots of other parameters and results from the two-compartment model can be found in the Supplementary Material.

**TABLE 3:** Inter-session and inter-visit mean grey matter values of CBF (ml/100 g/min), Texch (ms), ATT (ms), and ITT (ms) resulting from the extended model.

Parameter	Visit 1		Visit 2	
	Session 1	Session 2	Session 1	Session 2
Texch	227.9 ± 37.9	224.7 ± 49.6	239.7 ± 53.5	234.1 ± 53.1
CBF	63.6 ± 11.2	66.0 ± 8.8	65.8 ± 11.8	66.7 ± 6.1
ATT	1,187 ± 160.5	1,218 ± 137.5	1,175 ± 143.9	1,190 ± 148.8
ITT	310.3 ± 52.9	308.3 ± 56.4	327.7 ± 63.3	324.5 ± 62.8

Mean fluctuations measured within the Texch parameter, across the subjects, estimated by the extended model (14.3%) were less than those estimated by the two-compartment model (16.2%), although the difference was not significant (*P* = 0.321, Figure 10D).

## Discussion

In this study, we have demonstrated a non-invasive multi-TE ASL-based method with spatially resolved exchange time maps for the assessment of BBB integrity. An extended model was introduced accounting for within-voxel transit effects. Using this, a comparison between the two-compartment model and the extended model for estimation of Texch was assessed. For *in vivo* data, the mean grey matter Texch estimated by the extended model was 32.6% lower than the two-compartment model. This deviation can be attributed to additional estimation of ITT by the extended model, which attempts to separate the two mechanisms of intra-voxel transit and exchange. An investigation carried out for the Texch test-retest reproducibility of intra-session, inter-session and inter-visit showed a CoV of 6.6, 7.9, and 8.4%, respectively, showing that this technique has a potential to provide consistent results. The comparison between inter-visit differences estimated by the two models showed a trend that the extended model may result in lower fluctuations, reflecting an improved repeatability of Texch can be achieved by modelling the intra-voxel transit effects, although the results were not significant and should be further explored.

Our simulations performed with ITT = 200 ms showed that the two-compartment model, not taking into account the intra-voxel transit effects, resulted in more errors for all three estimated parameters – CBF, ATT, and Texch (Figure 3). The results provide an interesting interplay of all three parameters with ITT. The CBF errors showed that it is overestimated if ITT is not considered in the estimation process, which is in line with the reported literature (Alsop and Detre, 1996). Interestingly, CBF was still found to be overestimated with the extended model, although the error was much smaller, and could be considered as a limitation of the model. ATT estimated with the two-compartment model showed a trend of under-estimation, which explains the corresponding higher estimated Texch relative to the true value. The importance of inclusion of the additional delay time was further highlighted by the data generated with different ITT values. An ITT value of as low as 100 ms showed an overestimation of Texch, at a variable level, for the whole range of real Texch. Furthermore, our results showed that the fast exchange dynamics reflected by the lower Texch values (<300 ms) – which would be associated with a disrupted BBB in a disease – are the most affected and showed the highest over-estimation. On the other hand, the slow exchange dynamics reflected by the higher Texch values – which would be associated with an intact and healthy BBB – tend to be less affected. This highlights an important issue that if the tissue transit effects are not modelled carefully, this may result in an incorrect over-estimation of fast exchange times. This may lead to low sensitivity of the technique to differentiate a disrupted BBB from a healthy one.

Additionally, in our simulations, we observed errors appearing in a pattern of streaks around the TI values used (Figure 3). This could be explained by the Hadamard encoding scheme used, where the difference between two consecutive TIs is strictly equal to the bolus duration, meaning that there is no overlap of bolus between two adjacent TIs. The Bayesian estimation approach does not cope well with this feature of Hadamard encoding. An artificially extended bolus duration to emulate dispersion effects can be used to reduce these bandings, although this cannot completely resolve this issue. This can be seen in the ATT error for an extended bolus duration of ∼15%, where this streak pattern of error still appears, indicating poor estimation due to the absence partial bolus delivery between the two consecutive TIs.

The *in vivo* results showed a mean grey matter Texch value of 228 ± 38 ms, which is closer to reported BBB permeability values evaluated with PET (Raichle et al., 1983). The comparison of the estimated Texch values from the two models showed that the two-compartment model estimated a 50% higher Texch relative to the extended model. This reflects that such a difference can lead to a potentially low sensitivity of the method to detect BBB in pathologies. Furthermore, the extended model offers an estimation of the ITT parameter as well, further providing insight into the dynamics of vessels other than bigger arteries, offering an opportunity to explore the subject-specific and disease-specific changes in the brain microvasculature. Our results demonstrated a mean grey matter ITT value of 310 ± 53 ms, which is in line with the reported literature value (Li et al., 2005).

The two models considered in this study achieved similar goodness of fit. However, it is worth mentioning that when no intra-voxel transit is considered, that is at ITT = 0, the two models are the same. The fact that the extended model returns a positive value of ITT and the free energy value is quite close between the two models implies that there is some support for the extra complexity in the extended model explaining the data better.

The test-retest results from our study revealed good reproducibility of the Texch measure. The intra-session, inter-session and inter-visit CoV was 6.6, 7.9, and 8.4%, respectively. On the other hand, the inter-session and inter-visit CoV of CBF was found to be in the range of 11–14%, which is slightly higher than the reported reproducibility studies (Chen et al., 2011; Mutsaerts et al., 2014; Sousa et al., 2014). We observed that in two subjects, the inter-session data showed notably low ASL signal (difference of a factor of 2) in the decoded images. This presumably could have resulted due to lower labelling efficiency or inconsistent positioning of labelling slab, resulting in comparatively lower estimated CBF, which in turn reduced the reproducibility of the CBF parameter as a whole (Supplementary Figures 1, 2 show a scatter plot of CBF with 10 and 8 subjects, respectively). On the contrary, Texch parameter estimation was not strongly affected (Figure 10). This indicates the robustness of Texch assessment and relative independence on labelling efficiency, as estimation of this parameter largely depends on T2 decay, which is identical for different values of labelling efficiency. However, it stresses the importance of reproducible slice positioning, as the overall sensitivity will be greatly reduced for incompletely labelled blood magnetization. Moreover, higher variance in CBF could also be explained by the complexity of the extended model, which has more degrees of freedom and estimates more parameters.

Moreover, we computed fluctuations within the inter-visit Texch, estimated using the two-compartment model and the extended model, and compared the differences. The two-compartment model produced 13.3% more fluctuations relative to the extended model, although the difference was not significant *(P* = 0.321). This difference further increases to 22.4%, *P* = 0.207 if we analyse data with eight subjects, without including the two subjects that showed CBF error discussed earlier. There might be a trend in the data, but further studies with larger datasets are needed to evaluate this.

The study protocol was designed to include two different bolus durations with which all four parameters could be efficiently extracted. The first protocol uses a shorter bolus duration and estimates the inflow of the labelled bolus for ATT estimation. The second bolus – with a longer duration – was used to target the later tissue phase to capture the exchange dynamics. We previously showed that combining parallel imaging (CAIPIRINHA sampling) with such a protocol resulted in a scan time of less than 6 min (Mahroo et al., 2021). This offers a possibility to test and employ this non-invasive MRI technique in clinical trials for BBB integrity analysis.

The study shows a reliable and robust Texch parameter estimation, but there are several limitations that must be addressed. The model complexity already discussed above shows a trade-off between an improved and reliable estimation of Texch and an increased variation in the CBF parameter. ITT incorporation could lead to an improved sensitivity of the technique to detect changes between the healthy and the disrupted BBB by separating the intra-voxel transit effects. This could bring a meaningful difference in a clinical setting as the technique is completely non-invasive. On the other hand, the higher variation in the CBF parameter is still in an acceptable range of 11–14%. This could be reduced by splitting the estimation process, fitting CBF and ATT in one step, and using the estimated values to extract Texch and ITT values in the next step. The sensitivity of the ASL technique to improper shimming and dependence on the positioning of labelling slab should also be kept in mind, as this may lead to a substantial difference in the ASL signal measured. Finally, the technique needs to be evaluated in a larger cohort of healthy and diseased datasets to evaluate the sensitivity to detect the changes in the BBB. Comparative studies with other ASL-based techniques (Wang et al., 2007; Wells et al., 2009; St Lawrence et al., 2012; Lin et al., 2018; Shao et al., 2019) and contrast-agent-based methods (Montagne et al., 2015; Dickie et al., 2019; Varatharaj et al., 2019) could provide a valuable understanding of the underlying mechanisms each technique is probing.

## Conclusion

Multi-TE ASL has the potential to assess exchange dynamics with good repeatability. The extended model considering the intra-voxel transit effects showed a 32.6% lower Texch compared to the two-compartment model. Separately modelling the non-exchanging ITT phase may help us better investigate pathologies of distinct underlying causes, such as vessel obstruction and disrupted BBB. Being a water-based non-invasive MRI method, multi-TE ASL shows a potential to act as an imaging biomarker for neurovascular diseases.

## Data Availability Statement

The data analysed in this study is subject to the following licenses/restrictions: Participants of this study did not agree for their data to be shared publicly. Requests to access these datasets should be directed to AM, amnahmahroo@gmail.com.

## Ethics Statement

The studies involving human participants were reviewed and approved by the Ethics Committee of the University of Bremen, Bremen, Germany. The patients/participants provided their written informed consent to participate in this study.

## Author Contributions

AM processed simulated and *in vivo* data, conducted data analysis, and wrote the manuscript. MB developed the extended model. AM and JH collected *in vivo* data. MCh and MCr provided software related support for the analysis. MG and N-JB provided support for MR sequence and protocol. MG, MCr, HM, and AM discussed results and revised manuscript. All authors edited and revised the manuscript and approved final submission.

## Conflict of Interest

MG is the CEO of mediri GmbH. The remaining authors declare that the research was conducted in the absence of any commercial or financial relationships that could be construed as a potential conflicts of interest.

## Publisher’s Note

All claims expressed in this article are solely those of the authors and do not necessarily represent those of their affiliated organizations, or those of the publisher, the editors and the reviewers. Any product that may be evaluated in this article, or claim that may be made by its manufacturer, is not guaranteed or endorsed by the publisher.
