# Supplementary material for: Robust Multi-TE ASL-Based Blood–Brain Barrier Integrity Measurements
Source: Front Neurosci. 2021 Dec 3;15:719676. doi: 10.3389/fnins.2021.719676 (PMC8678075; doi:10.3389/fnins.2021.719676)
Supplement: Supplementary file 1 [file Data_Sheet_1.docx]

Supplementary Material

***Supporting Information Table S1:*** Parameter values used for simulations.

| **Parameter** | **Units** | **Value** |
| --- | --- | --- |
| T1 blood | ms | 1584* |
| T1 tissue | ms | 1331 |
| T2 blood | ms | 165 |
| T2 tissue | ms | 80 |
| CBF | ml/100g/min | 60 |
| M0 blood | a.u. | 1 |
| M0 tissue | a.u. | 1 |
| alpha |  | 0.85 |
| lambda |  | 0.9 |

*(Atlas, 2009)

***Supporting Information Table S2***: Intra-class Correlation (ICC) and Coefficient of variation (CoV) values of Texch, CBF, ATT and ITT for intra-session, inter-session and inter-visit for eight subjects (n=8). Data from two subjects was affected presumably by inefficient labelling and resulted in significantly reduced estimated CBF. Repeatability analysis after excluding the respected two subjects is shown in the table. It can be seen that the CBF parameter showed improvement for both ICC score and & %CoV measures.

| **Parameter** | **Session** | **ICC** | **P-value** | **% CoV** |
| --- | --- | --- | --- | --- |
| **Texch** | Intra-session | 0.788 | 0.003 | 7.7 |
|  | Inter-session | 0.927 | <0.001 | 4.6 |
|  | Inter-visit | 0.815 | 0.005 | 6.3 |
| **CBF** | Intra-session | 0.864 | 0.002 | 2.6 |
|  | Inter-session | 0.309 | 0.232 | 6.1 |
|  | Inter-visit | 0.050 | 0.455 | 5.4 |
| **ATT** | Intra-session | 0.988 | <0.001 | 1.2 |
|  | Inter-session | 0.967 | <0.001 | 2.1 |
|  | Inter-visit | 0.950 | <0.001 | 2.9 |
| **ITT** | Intra-session | 0.988 | <0.001 | 1.8 |
|  | Inter-session | 0.964 | <0.001 | 3.1 |
|  | Inter-visit | 0.874 | <0.001 | 5.1 |

***Supporting Information Table S3****:* Reproducibility results obtained using two-compartment model for intra-session, inter-session and inter-visit ICC and CoV for Texch, perfusion and ATT.

| **Parameter** | **Session** | **ICC** | **P-value** | **% CoV** |
| --- | --- | --- | --- | --- |
| **Texch** | Intra-session | 0.950 | <0.001 | 5.6 |
|  | Inter-session | 0.853 | <0.001 | 7.4 |
|  | Inter-visit | 0.699 | 0.0072 | 9.4 |
| **Perfusion** | Intra-session | 0.952 | <0.001 | 3.5 |
|  | Inter-session | 0.0004 | 0.4995 | 11.0 |
|  | Inter-visit | -0.718 | 0.9771 | 14.5 |
| **ATT** | Intra-session | 0.973 | <0.001 | 1.7 |
|  | Inter-session | 0.949 | <0.001 | 2.5 |
|  | Inter-visit | 0.913 | <0.001 | 3.3 |

***Supporting Information Table S4****:* Inter-session and inter-visit mean gray matter values of CBF (ml/100gm/min), Texch (ms), ATT (ms), and ITT (ms) resulting from two-compartment model.

| **Parameter** | **visit 1** | | **visit 2** | |
| --- | --- | --- | --- | --- |
|  | **session 1** | **session 2** | **session 1** | **session 2** |
| **Texch** | 342.0 ± 85.3 | 334.6 ± 94.3 | 361.5 ± 104.1 | 353.9 ± 103.5 |
| **CBF** | 67.8 ± 12.0 | 70.4 ± 10.2 | 70.3 ± 13.0 | 71.4 ± 7.6 |
| **ATT** | 1179 ± 164 | 120.8.8 ± 139.9 | 1163.8 ± 152 | 1180.6 ± 152.1 |

***Supporting Information Table S5****:* Mean free energy (log scale) within gray matter for the two models.

| **Sub** | **Two-compartment**  **Model** | **Extended Model** |
| --- | --- | --- |
| 1 | 9.08 | 9.14 |
| 2 | 9.21 | 9.31 |
| 3 | 8.69 | 8.76 |
| 4 | 8.55 | 8.63 |
| 5 | 8.00 | 8.03 |
| 6 | 8.32 | 8.35 |
| 7 | 8.36 | 8.42 |
| 8 | 8.32 | 8.37 |
| 9 | 8.56 | 8.61 |
| 10 | 8.78 | 8.81 |
| mean | 8.59 ± 0.37 | 8.64 ± 0.38 |


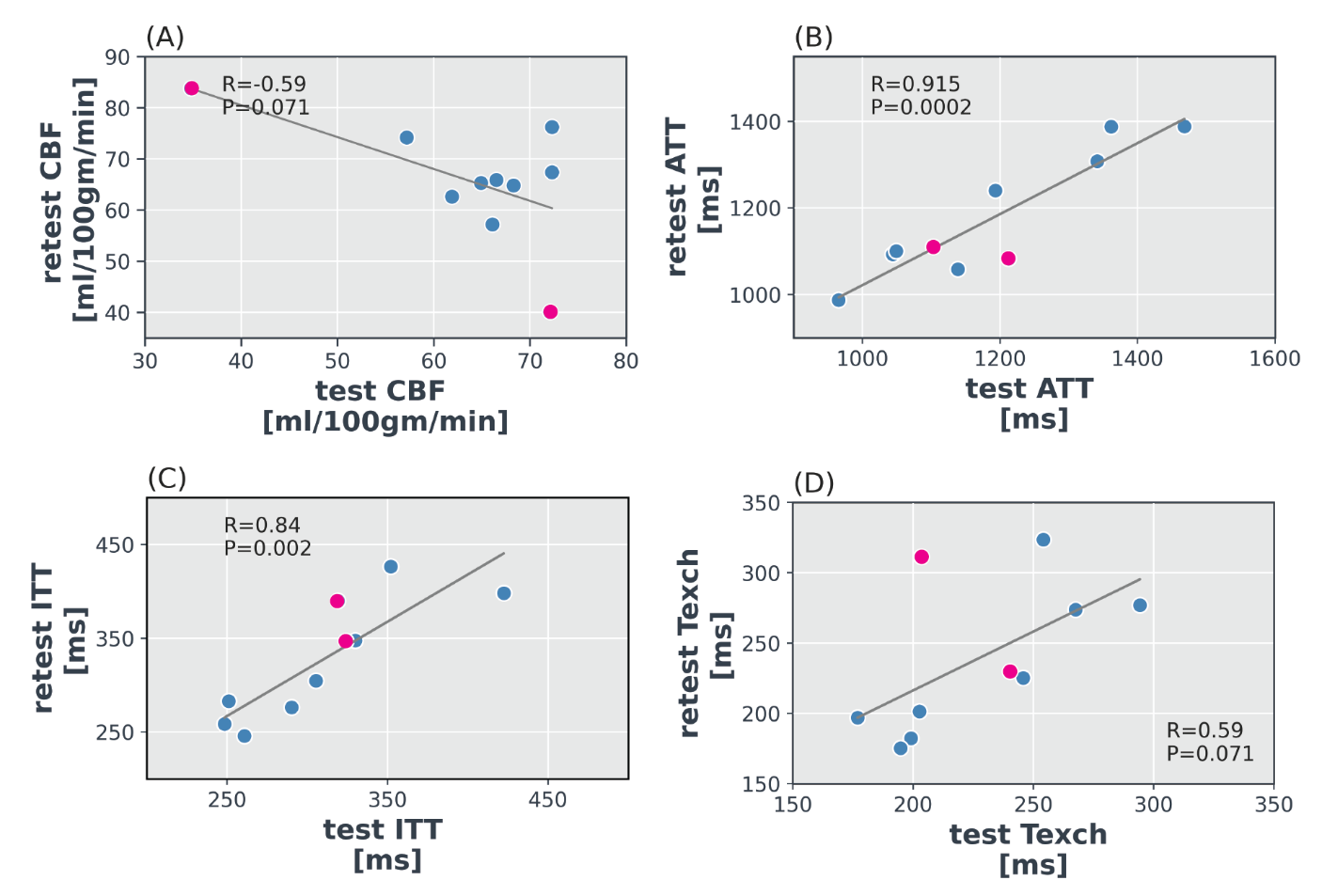


***Supporting Information Figure S1***: Inter-visit scatter plots of (A) CBF, (B) ATT, (C) ITT and (D) Texch are shown from all ten subjects. Data points from the two subjects which showed differences in ASL subtracted data are shown in pink.


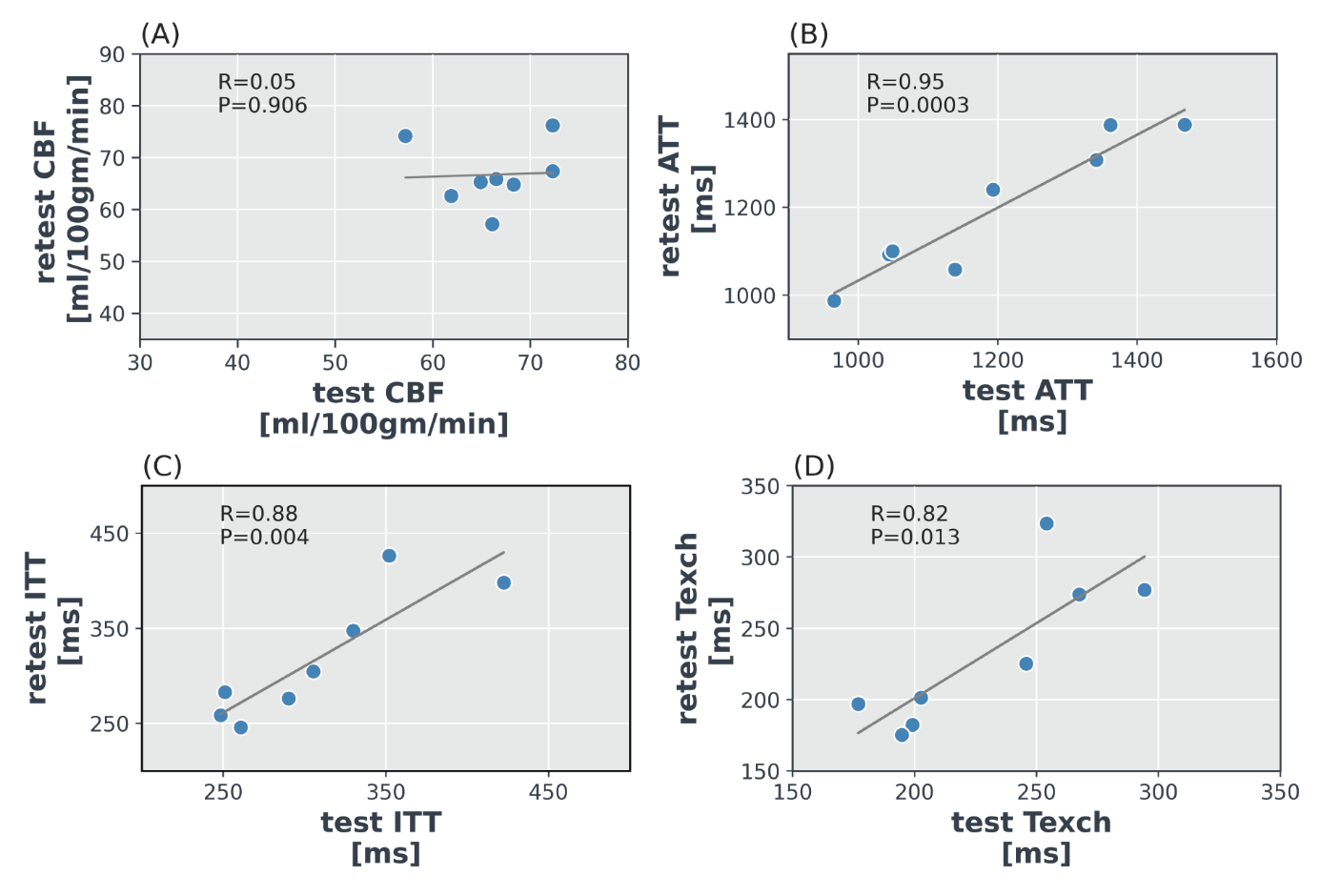


***Supporting Information Figure S2***: Inter-visit scatter plots of (A) CBF, (B) ATT, (C) ITT and (D) Texch are shown from eight subjects after excluding the two subjects with labelling efficiency issues.


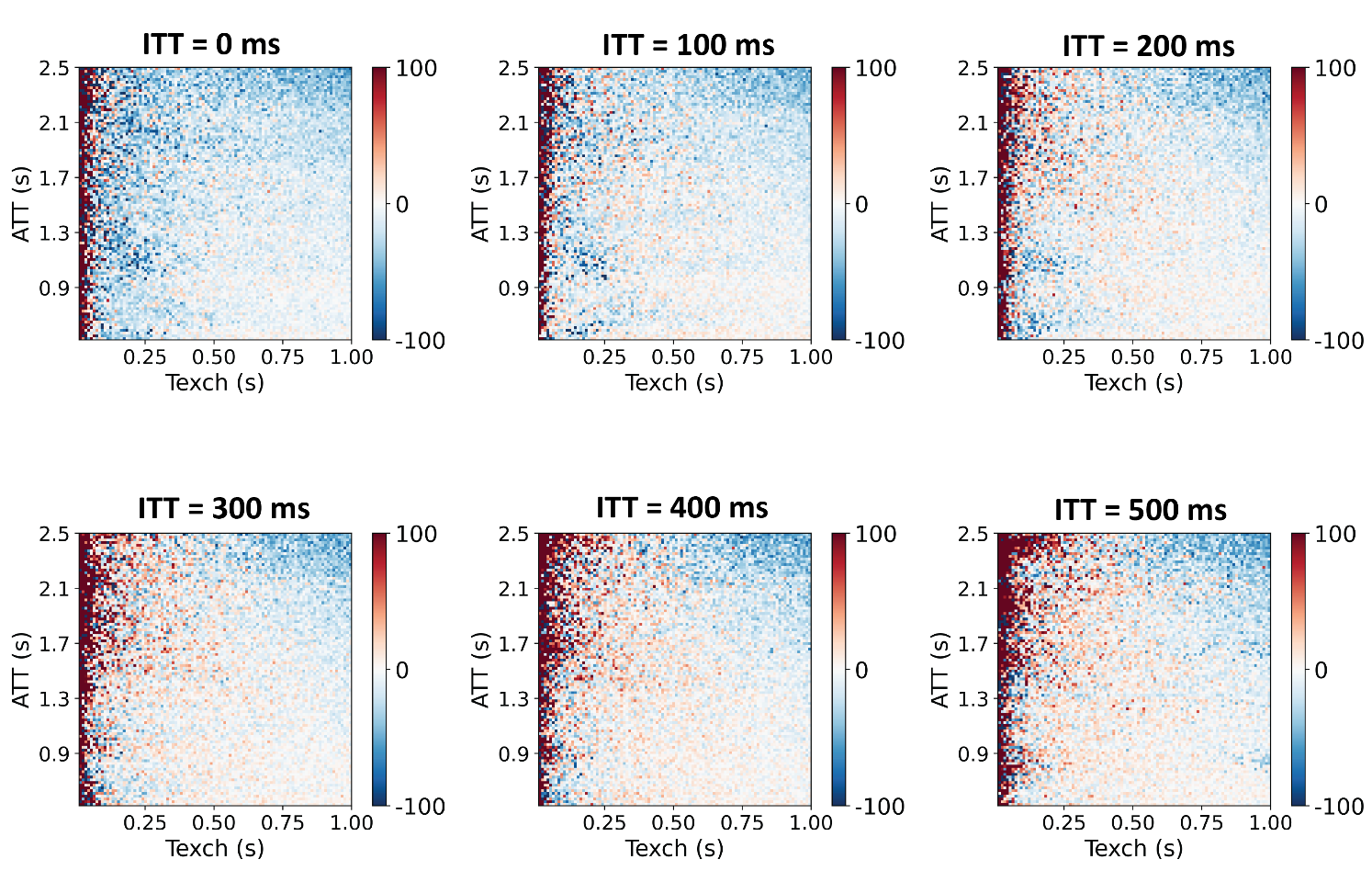


***Supporting Information Figure S3***: Texch percentage deviation error resulting from the extended model. Test cases were generated with variable ITT values.


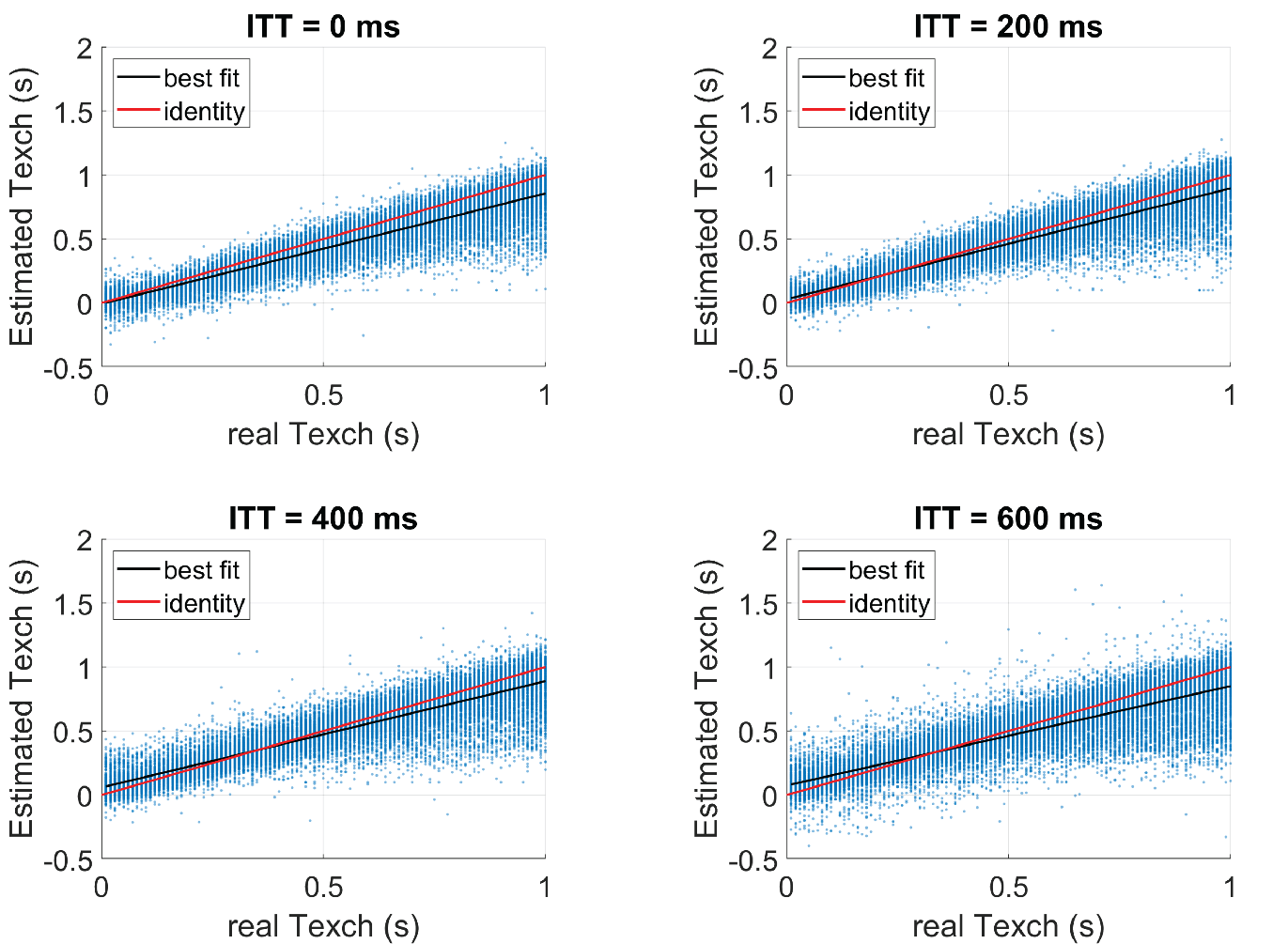


***Supporting Information Figure S4***: Identity line plots for Texch parameter estimated with the extended model. Test cases are generated with different values of ITT.


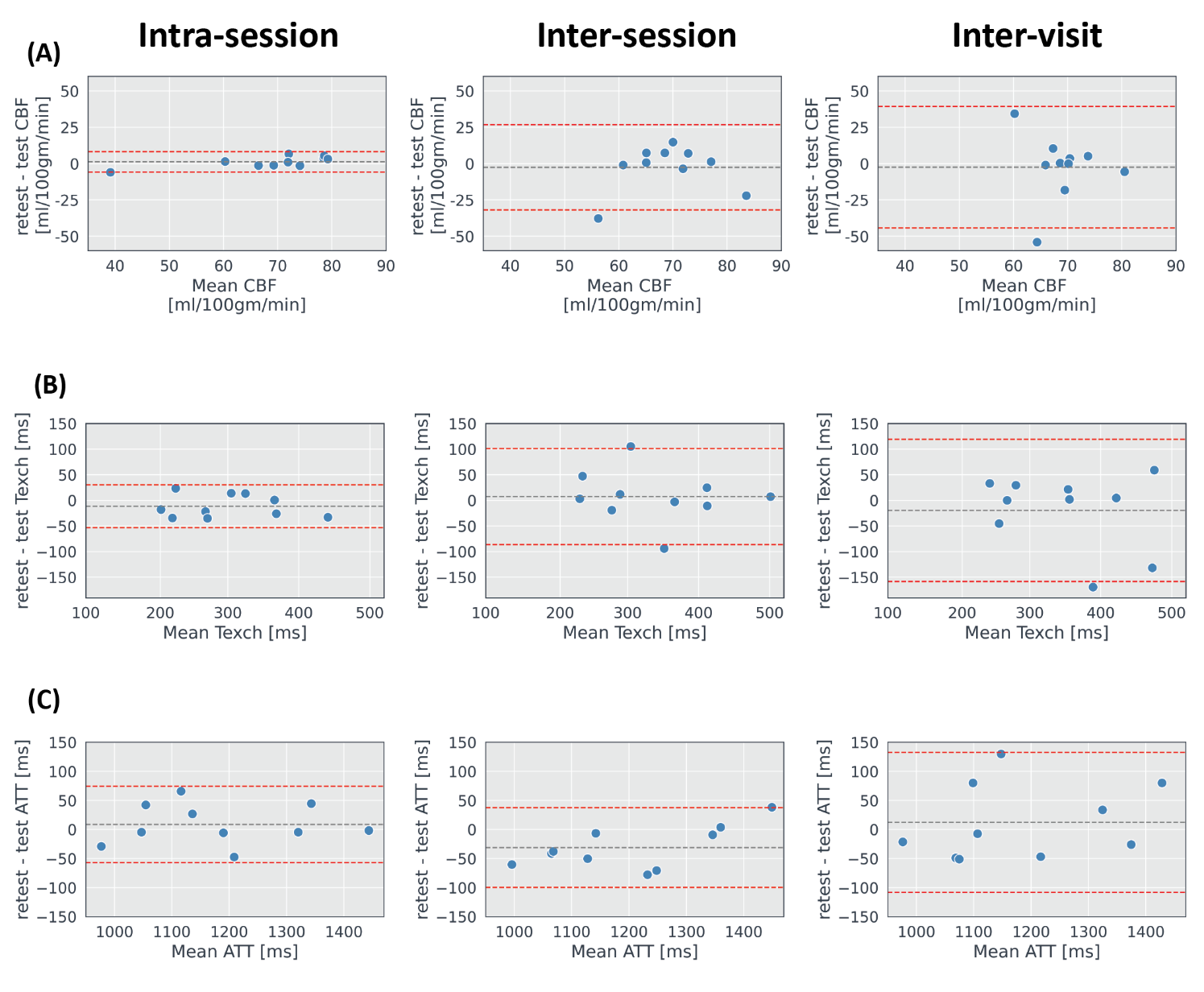
***Supporting Information Figure S5***: Bland-Altman Plots. Plots for CBF (A), Texch (B), and ATT (C) showing the spread of the data for intra-session, inter-session and inter-visit estimated with a two-compartment model.


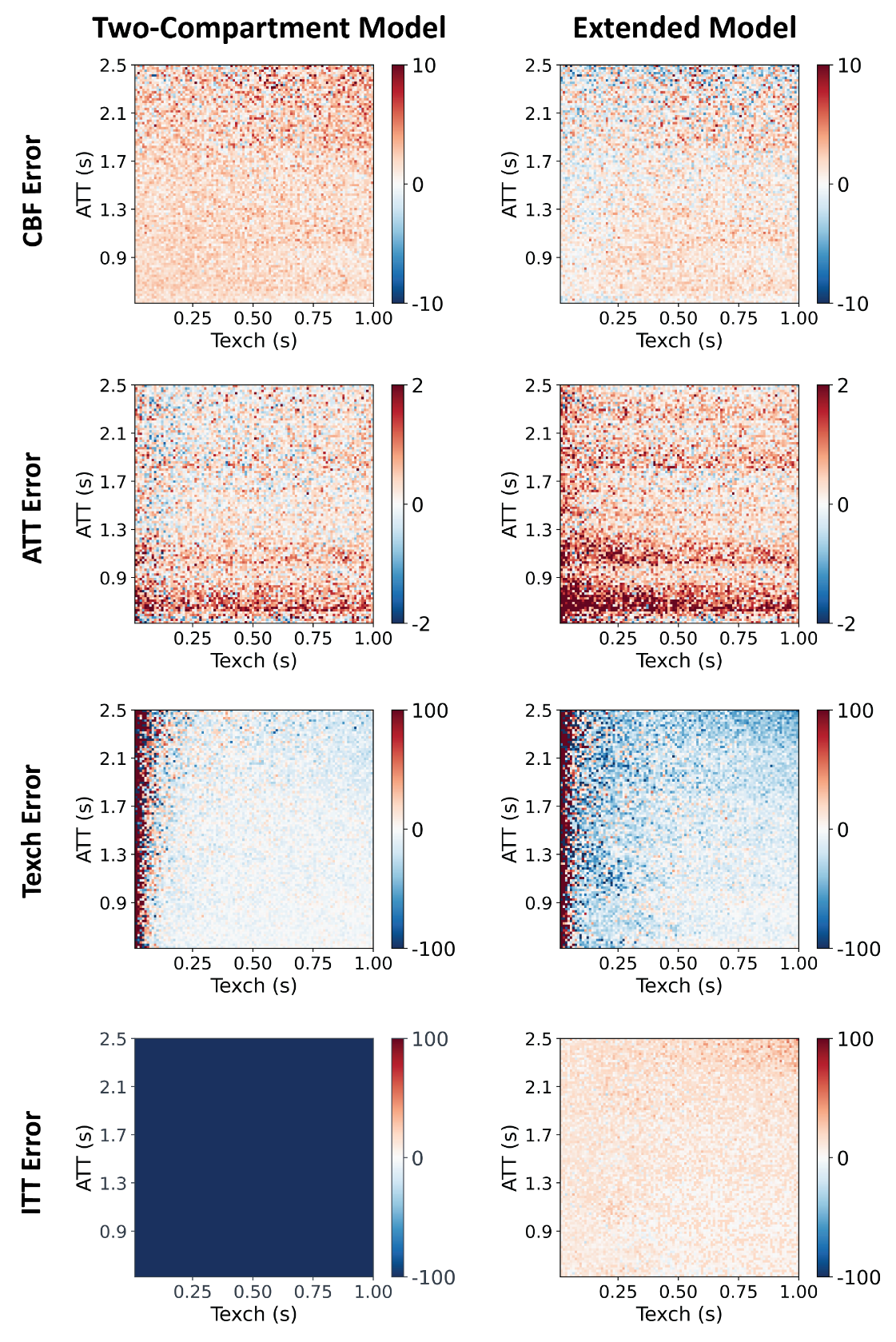


***Supporting Information Figure S6****:* Comparison of the two-compartment model and the extended model for ITT=0. Error in terms of percentage deviations of the estimated parameters is shown. The two-compartment model estimates Texch well while the extended model, assuming intra-voxel transit, shows a trend of underestimation for earlier Texch values. It also shows slightly higher ATT error, but estimates CBF with lower error in comparison to the two-compartment model.
